# Revision of the Neotropical Obscure Genus *Ebenia* Macquart 1846 (Diptera, Tachinidae, Dufouriini)

**DOI:** 10.1007/s13744-024-01156-3

**Published:** 2024-06-14

**Authors:** Marcelo Domingos de Santis

**Affiliations:** Zoologisches Forschungsmuseum Alexander Koenig, Leibniz-Institut zur Analyse des Biodiversitätswandels, Bonn, Germany

**Keywords:** Diversity, Host infection strategies, Neotropical Region, Redescription, Taxonomy

## Abstract

The Neotropical genus *Ebenia* Macquart, 1846, is a member of the tribe Dufouriini (Dexiinae), and before the current work, comprised four species, viz. *E. claripennis* Macquart 1846, *E. fumata* (Wulp, 1891), *E. neofumata* Santis & Nihei, 2022 and *E. trichopoda* (Wulp, 1891). The present taxonomic revision results in a new generic synonymy: *Comyops* Wulp, 1891 **syn. nov.** of *Ebenia*. The following two new combinations result from this act: *E. nigripennis* (Wulp, 1891) **comb. nov.** and *E. striaticollis* (Wulp, 1891) **comb. nov.** both originally described in *Comyops*. In addition, the species originally described as *Homodexia spinosa* Bigot, 1889 is moved from its current placement in *Thelairodes* Wulp, 1891 to *Ebenia* as *Ebenia spinosa* (Bigot, 1889) **comb. nov.** A new specific synonymy is proposed for this last species: *Morinia trichopoda* Wulp, 1891, previously treated as a valid species of *Ebenia*, becomes a **junior synonym** of *E. spinosa*. All valid species are redescribed and photographed with the first description and illustration of the male terminalia for *E. claripennis*, *E. neofumata* and *E. nigripennis* and female terminalia for *E. spinosa*. Additionally, lectotype fixations are made for *E*. *nigripennis* and *M. trichopoda*. Finally, an updated diagnosis for the genus *Ebenia* and a key to the six known species are provided.

## Introduction

The tachinid tribe Dufouriini is placed in Dexiinae and is currently known from all regions of the world (Santis & Nihei 2022). This tribe is composed of five genera: *Chetoptilia* Rondani, 1862; *Comyops* Wulp 1891; *Dufouria* Robineau-Desvoidy, 1830; *Ebenia* Macquart 1846a, b and *Rondania* Robineau-Desvoidy, 1850. Of those genera, only *Comyops* and *Ebenia* are from the Neotropical region. Although the overall trend of tachinids from this region is being poorly known (see, e.g., Santis 2021, 2022), both genera are an exception due to the redescriptions of some of its species amde by Thompson (1963). After the works of Townsend (1939), which placed *Comyops* and *Ebenia* in the former tribe Ebeniini together with taxa currently in Palpostomatini (Tachininae) and Voriini (Dexiinae), Thompson (1963) was the first to hypothesize the possible close relationship between these genera. Additionally, Thompson (1963) also noted that the male genitalia of *Comyopsis* resemble those of *Dufouria nigrita* (Fallén, 1810), as figured by Verbeke (1962: Fig. 1 of plate X).

The views of Thompson (1963) were confirmed by the molecular phylogeny of Stireman et al. (2019) that recovered an undetermined species of *Ebenia* in a clade that clustered it with genera such as *Dufouria* and *Rondania*, both dufouriines. In addition, Stireman et al. (2019) Dufouriini was recovered as paraphyletic. Thus, the precise limits of Dufouriini and the relationships among these taxa remained unclear. These issues were clarified by the morphological phylogeny of Dufouriini by Santis & Nihei (2022), in which this tribe was recovered as monophyletic and conclusively recovered the following phylogenetic relationships: (*Rondania* (*Chetoptilia* (*Dufouria* (*Ebenia* + *Comyops*). Even though the phylogenetic placement of *Ebenia* is well known, the taxonomic delimitation of this genus is far from clear, with its current species *E. claripennis* Macquart 1846a, b, *E. fumata* (Wulp 1891), *E. neofumata* Santis & Nihei 2022 and *E. trichopoda* (Wulp 1891) not appearing in any key or comparative analysis. In consonance with that, the three host records for this genus are all Chrysomelidae (Coleoptera) based on undetermined species: Janzen & Hallwachs (2005) for *Sceloenopla scherzeri* (Baly, 1858), Cuignet et al. (2008) for *Chelymorpha alternans* Boheman, 1854 and *Spaethiella marginata* (Champion, 1893); similarly, the phylogeny of Stireman et al. (2019) could not determine the species of *Ebenia* sampled in their work. Interestingly, a peculiarity of the members of this tribe is their hosts being adult beetles and their female terminalia being modified into different forms (Santis & Nihei 2022) to achieve the introduction of their larvae into those beetles, and particularly, *Ebenia* females that bears the sternite 8 as a cone–shaped structure.

The aim of this study is to provide a comprehensive revision of the species of genus *Ebenia*, and to clarify its generic and specific limits. All species were redescribed and photographed, in addition to the first description and illustration of the male terminalia for *E. claripennis*, *E. neofumata* and *E. nigripennis* and female terminalia for *E. spinosa*. Additionally, lectotype fixations are made for *E. nigripennis* and *M. trichopoda*.

## Material and Methods

The examined material from each institution, as well as the mode of access of the material (loan, photos, visit), are indicated as follows:

ARC – Arthropod Research Collection, Michigan State University, Michigan, USA – loan;

MNCR, Museo Nacional de Costa Rica [formerly Instituto Nacional de Biodiversidad – INBio], Santo Domingo de Heredia, Costa Rica – loan;

MNRJ, Museu Nacional, Universidade Federal do Rio de Janeiro, Rio de Janeiro, Brazil – visit and loan; MZSP, Museu de Zoologia da Universidade de São Paulo, São Paulo, Brazil – visit and loan;

NHMUK, Natural History Museum, London, UK – visit;

USNM, Smithsonian National Museum of Natural History, Washington, D.C., USA – photos.

Additional acronymous in the text: OMNH – Oxford University Museum of Natural History, Oxford, UK. The label data are presented within quotation marks for each label, with forward slashes indicating line breaks and semicolons separating different labels. Morphological terminology follows Cumming & Wood (2017).

Photographs of the pinned specimens were taken using a Canon EOS 5DS R digital camera for the material deposited at NHMUK, a Leica MC170 HD digital camera attached to a Leica MZ16 stereomicroscope for the MNRJ and MZSP material, and an Olympus E-M5 digital camera for the material deposited at USNM. The images were subsequently stacked (merging different focal planes into one image) with the software Helicon Focus 7.5.8 and edited in Adobe Photoshop CC 2019. Illustrations were made using a camera lucida attached to a Leica MZ16 stereomicroscope, and edited and arranged in Adobe Illustrator CC 2019. To digest tissues and clear structures, the last abdominal segments were put into a glass tube containing a 10% KOH solution and heated in boiling water for 5 min, neutralized in a 5% acetic acid solution, and rinsed in distilled water. After examination, the dissected parts were placed in glycerin inside a plastic microvial pinned with the source specimen.

## Taxonomy

Genus *Ebenia* Macquart, 1846a, b

*Ebenia* Macquart, 1846a: 299 (also published separately in 1846b: 171). Type species: *Ebenia claripennis* Macquart, 1846a, b, by original designation (Brazil).

*Comyopsis* Townsend, 1919: 176. Type species: *Comyopsis fumata* Townsend, 1919, by original designation. Synonymy given by Santis & Nihei (2022).

*Comyops* Wulp, 1891: 213, in key (1891: 262, description). Type species: *Comyops nigripennis* Wulp, 1891, by subsequent designation of Brauer and Bergenstamm, 1893. **New synonymy**.

**Remarks**. Macquart (1846a, b: 299) mentioned about *Ebenia* the following: “Le type de ce genre est du Brésil” [“The type of this genus is from Brazil”]. This statement is accepted as a type species designation for *Ebenia* of the single included species, *Ebenia claripennis* Macquart, from Brazil.

Included species:

*claripennis* Macquart, 1846a, b: 299. Holotype ♀: Brazil (NHMUK);

*fumata* (Wulp, 1891: 261) (*Morinia*). Holotype ♂: Mexico, Tabasco (NHMUK);

*neofumata* Santis & Nihei, 2022: 33 (*nomem novum* for *Comyopsis fumata* Townsend, 1919: 176). Holotype ♂: Nicaragua: Chinandega (USNM);

*nigripennis* (Wulp, 1891: 262) (*Comyops*). Lectotype ♂ (by designation herein) Mexico, Tabasco, Teapa (NHMUK) **comb. nov.**;

*striaticollis* (Wulp, 1891: 262) (*Comyops*). Holotype ♂: Mexico, Guerrero, Venta de Zopilote (NHMUK) **comb. nov.**;

*spinosa* (Bigot, 1889: 268) (*Homodexia*). Holotype ♂: Mexico (NHMUK) **comb. nov.**;

*Morinia trichopoda* Wulp, 1891: 261. Lectotype ♂ (by designation herein) : Mexico, Tabasco, Teapa (NHMUK) **new synonymy**

**References.** Brauer & Bergenstamm (1891: 381, redescription of *Comyops*); Brauer & Bergenstamm (1893: 183, designation of *C*. *nigripennis* Wulp, 1891 as type species of *Comyops*); Townsend (1936: 49, diagnosis of adults and immatures of “Ebeniini” including *Comyops*, *Comyopsis*, and *Ebenia*); Townsend (1939, redescription of: 156, *Comyops*; 157, *Comyopsis* and *Ebenia*); Guimarães (1971, catalogue of Neotropical Tachinidae: 109, *Comyops*, *Comyopsis*, and *Ebenia*); Janzen & Hallwachs (2005, host record of *Ebenia* spp. in larvae of *Sceloenopla scherzeri* (Baly, 1858)); Cuignet et al. (2008: host record of *Ebenia* spp. in larvae of *Chelymorpha alternans* Boheman, 1854 and *Spaethiella marginata* (Champion, 1893)); Wood & Zumbado (2010: 1372; 1392, *Ebenia* in key to Central American Tachinidae; 1403, comments about distribution and hosts); Evenhuis et al. (2016: 58, genus-groups names of Macquart) Stireman et al. (2019, *Ebenia* in molecular phylogenetic analysis of Tachinidae); O’Hara & Henderson (2020, world checklist of tachinid genera: 21, *Comyops*; 26, *Ebenia*); O’Hara et al. (2020, checklist of World Tachinidae: 91, *Comyops*; 93, *Ebenia*); Santis & Nihei (2022, *Comyopsis* as synonymous with *Ebenia*; morphological phylogenetic analysis of Dufouriini).

General characterization. Small, about 4.5 mm, blackish flies, with big eyes, almost touching on top, with arista long plumose, gena height very narrow in males, scutellum somewhat triangular and with abdomen mostly cylindrical.

Eyes with long setulae or scattered short setulae. Ocellar setae well developed, divergent and proclinate. Inner and outer vertical seta decussate and long. Arista long plumose. Fronto-orbital plate with uppermost frontal seta not reaching antennal insertion. Fronto-orbital plate with several setae around the antennal socketwihtout setae in male and with two proclinate and two reclinate orbital setae in females. Fronto-orbital plate in females about twice larger than males., parafacial bare. Genal dilation poorly developed. Lower facial margin not protruding, invisible in profile. Vibrissa arising at the level of lower facial margin, long and converging. Thorax. Postpronotal lobe with 2 setae. Notopleuron with 2 equal-sized setae. Intra-alar setae 1 + 2. Intra-postalar seta absent. Supra-alar setae 1 + 2. Postalar callus with 2 setae. Propleuron bare. Katepimeron setulose only on anterior region, with 1–3 setulae. Anepimeron with numerous long setae. Katepisternal setae 2. Anatergite setulose apically. Scutellum with one pair of basal, discal, lateral setae, and a pair of decussate apical setae; the apical one about 2x longer than the lateral. Postmetacoxal area membranous. Posterior spiracle with posterior lappet larger than anterior. Wing, with costal spine present. Cell r_4+5_ open at wing margin; length of opening shorter than crossvein r-m. Crossvein dm-cu sinuous. Vein M_1_ ending at wing margin close to tip, bent forward to R_4+5_, forming an angle slightly smaller than 90°, and convex after bend. Legs. Males with claws and pulvilli longer than tarsomere 5, shorter in females. Abdomen with syntergite 1 + 2 with at least 4 pairs of lateral marginal setae; tergite 3 with at least 4 pairs of lateral marginal setae and a pair of median marginal seta, tergite 4 and 5 with a row of marginal setae. **Male terminalia**. Tergite 6 undivided, about 1/5 length of syntergosternite 7 + 8. Syntergosternite 7 + 8 broad. Sternite 6 symmetrical. Anterior epandrial process undeveloped. Cerci not fused, narrow, and distally slightly tapered in posterior view. Ejaculatory apodeme fan-shaped. Hypandrial arm short, hypandrial apodeme poorly distinguishable, with narrow central plate. Bacilliform sclerite, long, rod-like. Epiphallus present, distally narrow, and fused with basiphallus. Pregonite and postgonite not fused; pregonite fully fused to each other; postgonite with anterior margin weakly sclerotized, with setulae on anterior margin. Basiphallus subrectangular, usually as long as postgonite. Extension of dorsal sclerite of distiphallus long, more than half of the length of dorsal sclerite; dorsal sclerite ventrally serrulated; granular zone absent.

### *Diagnosis*

In order to differentiate *Ebenia* from other Dufouriini genera, the following diagnosis is given: Arista long plumose. Postpedicel about 2x the combined lengths of scape and pedicel. Fronto-orbital plate with several setae on the antennal socket. Prosternum setulose or bare. Anepimeron with numerous long setae. Anatergite setulose apically. Wing with vein M_1_ ending at wing margin close to tip, bent forward to R_4+5_, forming an angle slightly smaller than 90°. Abdominal tergites without discal setae. *Male terminalia*. Tergite 6 undivided, about 1/5 length of syntergosternite 7 + 8. Syntergosternite 7 + 8 broad. Sternite 6 symmetrical. Sternite 5 with slightly developed lobules, setulose, with basal plate long and slightly curved; sensilla “*trichodea*” present on distal portion. Ejaculatory apodeme fan-shaped.

**Phylogenetic position.** One undetermined species of *Ebenia* was included in the molecular phylogeny of Stireman et al. (2019) and three species of *Ebenia* and one species of *Comyops* (here as **syn. nov.** of *Ebenia*) were included in the phylogenetic analysis of Santis & Nihei (2022). Both studies conclusively placed *Ebenia* in the tribe Dufouriini (Dexiinae), and the latter placed *Ebenia* + *Comyops* as sister group of the genus *Dufouria* Robineau-Desvoidy, and constituting a clade. This clade, named by Santis & Nihei (2022) as clade 15, is supported by four unambiguous synapomorphies: fronto-orbital plate with several setae on the antennal socket (43:1); male terminalia with phallapodeme with fan-shaped apex (126:1); distiphallus with ventral sclerite with dorsal projection (139:1) and distiphallus with a distal portion (144:1). Additionally, clade 16 comprises *Ebenia* and *Comyops* and is supported by one unambiguous synapomorphy: male terminalia with surstylus with lateral setae (119:1). It is also supported by three unambiguous homoplasies: antenna with postpedicel subcylindrical (5X the ratio of length to width) (54:0); antenna with plumose arista (55:2) and abdominal tergites with pruinosity only on anterior margin (93:0). *Comyops* possesses a single autapomorphy that differentiates it from *Ebenia*: facial ridge with setulae until the antennal insertion (58:1). *Ebenia* spp., that included *E. spinosa* (as *E.* undetermined sp), *E. claripennis* and *E. neofumata*, possesses a single autapomorphy, male terminalia with cerci with a globose expansion (114:1) and two homoplasies, first instar larva with segment IV without microtrichia (14:1) and vein R_4+5_ with dorsal setulosity beyond R base (87:1). Following the synonymy proposed herein, the synapomorphy of clade 16 it is an autapomorphy of the newly defined *Ebenia*.

**Distribution.** Mexico (Veracruz, Tabasco, Guerrero), Costa Rica (Guanacaste, Cartago), Nicaragua, Trinidad & Tobago and Brazil (Rio de Janeiro). In addition, based on the records of the CNC online database, some undetermined specimens of *Ebenia* further expands the distribution of this genus by the following countries: Venezuela (Aragua), Colombia (Valle Rio Anchicaya), Ecuador (Napo) and Bolivia (Cochabamba).

**Remarks.** Guimarães 1971: 216) listed *Hylemyia probata* Walker, 1861 from Mexico as an unrecognized species of Tachinidae. I examined the holotype of this species in NHMUK and it is in poor condition with only a portion of the thorax remaining. D.M. Wood examined the specimen in 1989 and labeled it as “*Ebenia*”, but I have concluded that a determination to genus, or even to subfamily, is not possible based on the remaining structures of the holotype.

## Key to Dufouriini Genera

The dufouriines, as recognized by Santis & Nihei (2022), are difficult to be succinctly characterized as many characters vary among its genera. Usually, dufouriines are small blackish tachinids with males bearing big eyes, almost touching on top, while females have eyes that are widely separated. In addition, females bear specialized terminalia that are particularly adapted to infect adult beetles utilizing different strategies and forms. To further assist the reader, a generalized description of the tribe is given: the head is always without orbital setae in males, parafacial is bare, facial carina absent, antennal axis at or below middle level of eye; lower facial margin not prominent, prementum shorter than head height. Thorax with bare prosternum, with two equal-sized notopleural setae, scutellum usually with strong apicals and basals; wing with cell R_5_ open, just closed at the wing margin, or long petiolate. Abdomen is ovalate to somewhat cylindrical, chaetotaxy is irregular, with variation in both numbers and strength of setae, it may be absent, with just setulae, as in *Rondania dorsalis* (Coquillett, 1902) or with numerous strong setae, as in *Dufouria chalybeata* (Meigen, 1824).

The following key is constructed in order to facilitate the recognition of dufouriini genera.

1 - Antennae with plumose or pubescent arista, wing with costal spine developed in various lengths, cell R_5_ open or just closed at the wing margin, abdomen shiny black or dark metallic, females with terminalia not exposed externally … 2.

- Antennae with pubescent or bare arista, wing without costal spine and cell R_5_ closed at the wing margin or petiolate, abdomen with grey and yellow/orange coloration, females with terminalia exposed externally with 6th and 7th segment tubular and posteriorly directed (see Fig. 19B of Santis & Nihei 2022) … *Rondania* Robineau-Desvoidy, 1850.

2 - Eyes with setulae, appearing in different degrees, fronto-orbital plate with several setae on the antennal socket, females with terminalia presenting tergite 8 fused with sternite 8 in a cone shape and posteriorly directed (Fig. 11B) … 3.

- Eyes bare, fronto-orbital plate without several setae on the antennal socket, females with terminalia presenting tergite 8 fused with sternite 8 in a peak shape and ventrally directed (see Fig. 20B of Santis & Nihei 2022) … *Chetoptilia* Rondani, 1862.

3 – Thorax with prosternum setulose or bare, anatergite setulose on upper region, abdomen without dense, erected and long setulae, without discal setae … *Ebenia* Macquart 1846a, b

- Thorax with prosternum bare, anatergite bare, abdomen with dense, erected and long setulae, with discal setae … *Dufouria* Robineau-Desvoidy, 1830.

## Key to *Ebenia* species

1 - Eyes with conspicuous setulae (Fig. 7b; 8b) … 2.

- Eyes bare, or practically bare, at most with very short and widely scattered setulae… 3.

2 - Thorax without pruinosity, wing smoky, abdomen with silvery pruinosity occupying just anterior margin of each tergite, about 1/5 of each segment laterally (Fig, 7a, c) … *E. nigripennis* **comb. nov.**

- Thorax with silver pruinosity, wing hyaline, abdomen with silvery pruinosity almost reaching posterior margin laterally on each tergite (Fig. 8a, c) … *E. striaticollis* **comb. nov.**

3 - Prosternum with setulae … 4.

- Prosternum bare … 5.

**Fig. 1:**
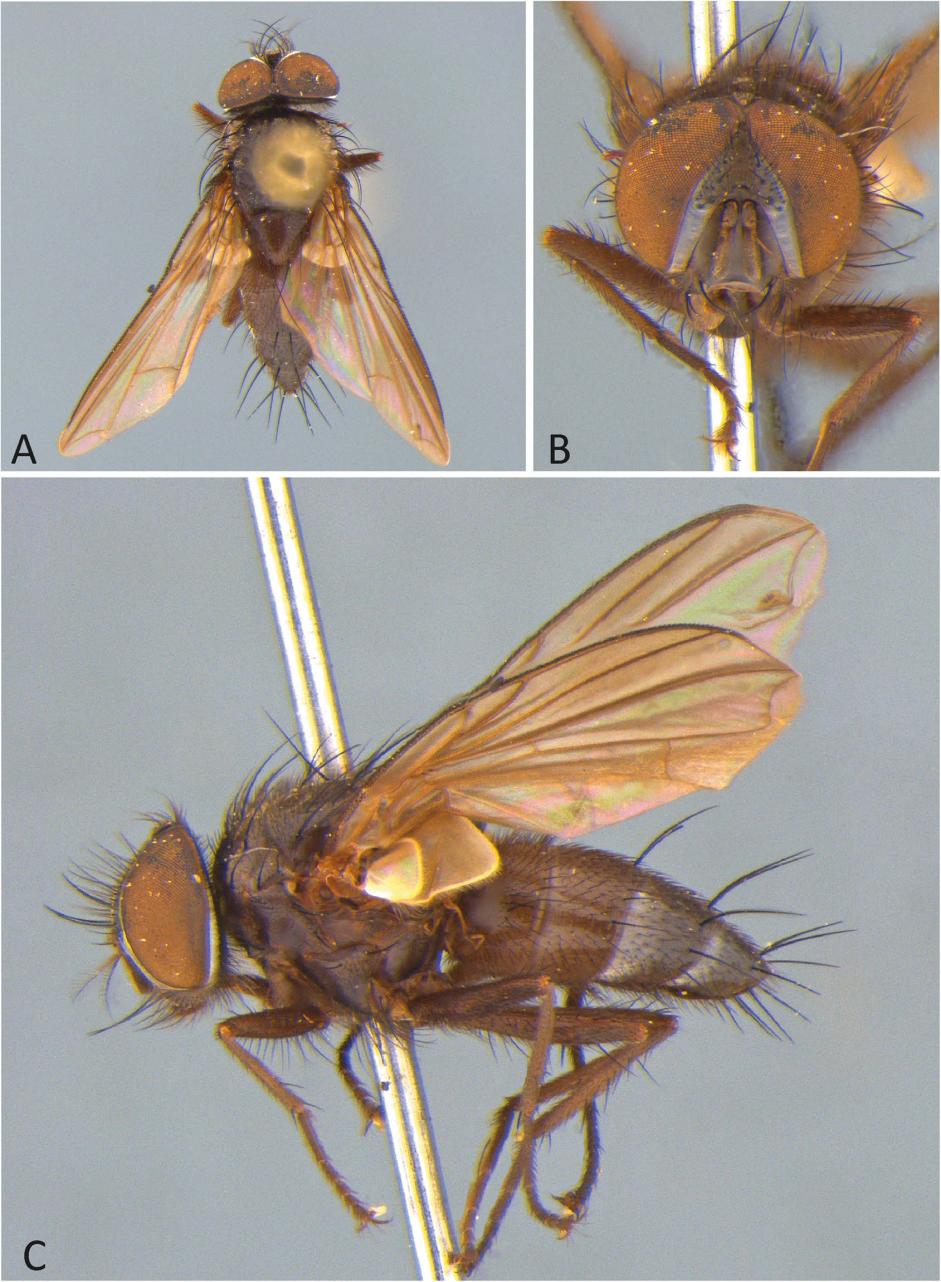
*Ebenia claripennis* Macquart 1846a, b, male from Rio de Janeiro, Brazil (MZSP). **A**. dorsal habitus. **B**. head, frontal view. **C**. lateral habitus

4 - Wing with vein R_4+5_ with setulae beyond or at the level of r-m dorsally, wing hyaline (Fig. 1a, c). … *E. claripennis.*

- Wing with vein R_4+5_ with setulae ending about ¼ to crossvein r-m dorsally, wing smoky (Fig. 3a, c) … *E. fumata.*

5 - Abdominal tergites without pruinosity, brownish black (Fig. 9a, c; 10 a, c) … *E. spinosa.*

- Abdominal tergites with silvery pruinosity, better visible on posterodorsal view, blackish (Fig. 4a, c) … *E. neofumata.*

***Ebenia claripennis*** Macquart 1846a, b

(Figs. 1 and 2).

**Fig. 2:**
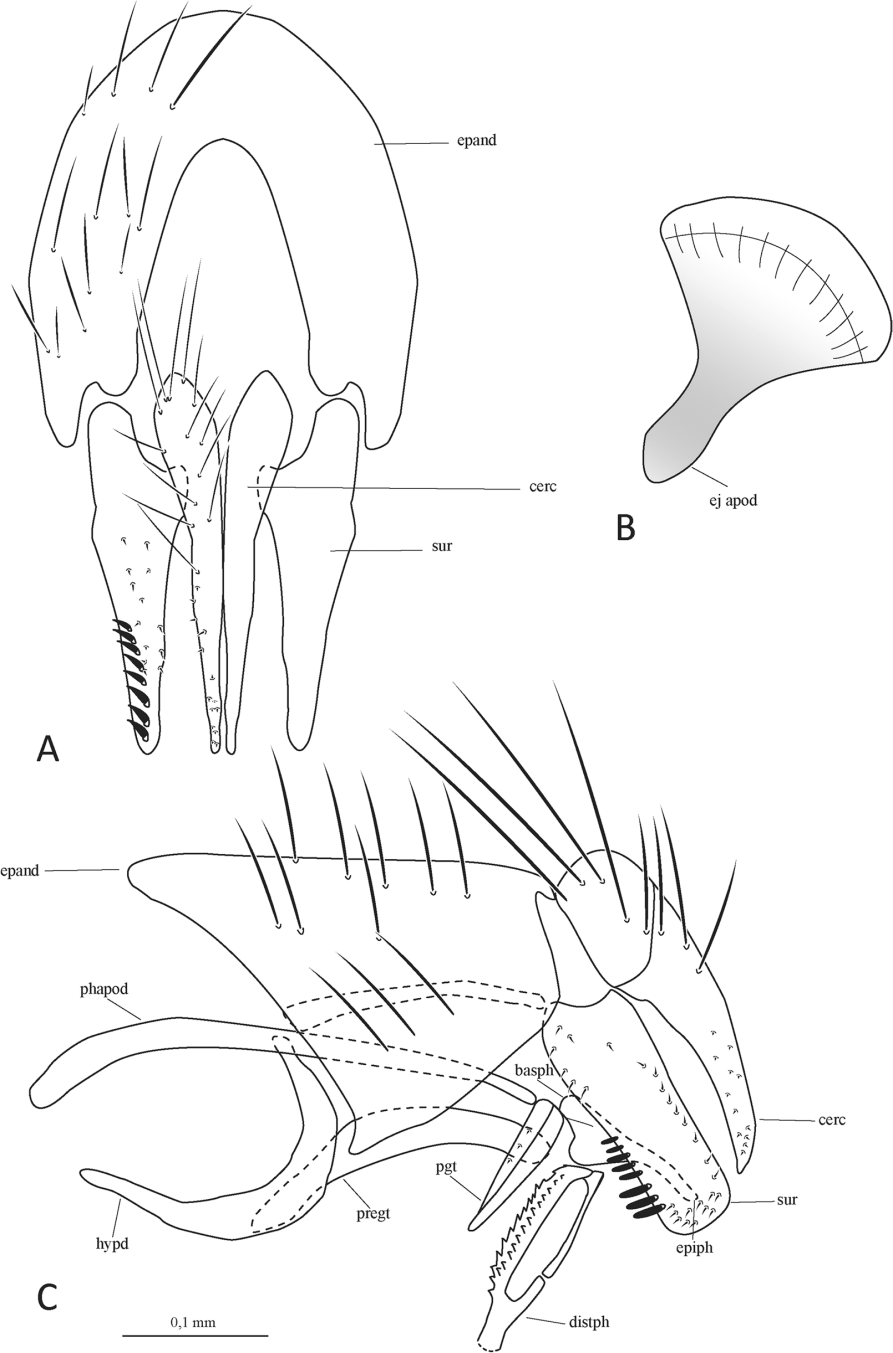
*Ebenia claripennis* Macquart 1846a, b, Veracruz, Mexico (ARC). Male terminalia. **A**. epandrium, cerci and surstylus, posterior view. **B**. ejaculatory apodeme. **C**. epandrium, cerci, surstylus, hypandrium, phallapodeme, basiphallus, epiphallus, distiphallus, pregonite and postgonite, lateral view. Abbreviations: basph = basiphallus; cerc = cercus; distph = distiphallus; ej apod = ejaculatory apodeme; epand = epandrium; epiph = epiphallus; hypd = hypandrium; pgt = postgonite; phapod = phallapodeme; pregt = pregonite; sur = surstylus

*Ebenia claripennis* Macquart 1846a: 299 (also published separately in b: 171). *References*. Brauer (1898: 515, diagnostic traits); Townsend (1893: 22, catalogue); Thompson (1963: 478, redescription of male; 480, description of first instar larva); Guimarães 1971: 109, catalogue); O’Hara et al. (2020: 93, checklist of World Tachinidae); Santis & Nihei (2022, phylogenetic analysis).

Described from an unspecified number of females from “Brésil” [Brazil] from the collection of “M. Bigot”, which is the private collection of Jacques-Marie-Frangile Bigot. Bigot’s collection is mostly in the NHMUK and a smaller fraction in the OUMNH; all tachinids are deposited at NHMUK (Crosskey 1971). For a complete explanation of the acquisition of Bigot’s collection by the NHMUK, the reader can consult Crosskey (1971).

At the NHMUK Diptera collection there is a single female that bears the usual label from Macquart that indicates a type specimen and includes the suffix “n.g., n.sp.” after the name [“Ebenia claripennis ♀”] and the additional label of Bigot indicating the specific name and the sex symbol towards the top, the type-locality on the bottom left [“Brésil”] and authority of the species at the bottom right [“Macq.”]. Although there is a label attached to this specimen that indicates that this is a “Holotype”, Macquart did not restrict the name-bearing type to a single specimen and no lectotype fixation has been published subsequently. Thus, the “holotype” in NHMUK is technically a syntype (see Recommendation 73F of the Code (ICZN 1999), “Avoidance of assumption of holotype”).

In the interests of nomenclatural stability and to restrict the name to a single specimen, female syntype in NHMUK is herein designated as lectotype of *Ebenia claripennis* Macquart 1846a, b.

The current combination for this species is *Ebenia claripennis* Macquart 1846a, b.

### Type material examined

Lectotype ♀: “Holo-/ type”; “Ebenia/ claripennis/ ♀. n. g. nov. sp.” [handwritten]; “Ebenia claripennis ♀/ Brésil Macq.” [handwritten]; “Ebenia/ claripennis Macq./ holotype ♀/ Brazil [handwritten]/ ex. Bigot Coll:/ B.M.1960–539.”

Lectotype in poor condition. Specimen molded, with head, legs and abdomen detached from pinned thorax.

### Additional examined material

MEXICO. Veracruz: Acayucan, 1 ♂, 23.x.1957, R. & R. Dreisbach (ARC); BRAZIL. Rio de Janeiro: Nova Friburgo, 26.4.1937, 1 ♂, S. Lopes col. (MZSP), Universidade Rural do Rio de Janeiro, 1 ♂, 24.v.1961, Deak col (MZSP).

**Diagnosis.** Eyes with very small and widely scattered setulae. Fronto-orbital plate dark silver pruinose. Postpedicel entirely dark brown. Facial ridge with setulae only at base. Prosternum with setulae. Thorax with silver pruinosity. Wing hyaline; vein R_4+5_ with setulae beyond the cross-vein r-m. Costal spine developed. Abdomen light brown with silvery pruinosity anteriorly on tergites 3 to 5. Male terminalia with surstylus bearing short spines laterally on posterior view.

Redescription of male.

**Coloration** (Fig. 1): Occiput with silver pruinosity. Head with dark silver pruinosity. Scape light brown and pedicel dark brown. Postpedicel dark brown. Arista dark brown, but proximal 1/5 light brown. Palpus tawny to yellowish. Labellum light brown, prementum shiny black. Scutum brownish, but presutural region and anterodorsal portion of postsutural region with brownish-silvery pruinosity; presutural region with five brownish-black vittae, the three central ones narrow and the two peripheral ones broad. Scutellum brownish. Wing hyaline. Tegula light brown, basicosta yellow. Halter yellowish-brown. Posterior spiracle light-brown. Legs brownish. Upper and lower calypters hyaline. Abdomen light brown with anterolateral silver pruinosity on tergites 3 to 5.

**Head** (Fig. 1): Vertex about 0.18 × head width in dorsal view. Width of parafacial, measured at distance between inner margin of eye and antennal insertion, 2 × height of gena. Postpedicel about 1.5 × the combined lengths of scape and pedicel. Frontal vitta narrowed dorsally. Eye about 0.8 × the head height. Gena about 0.12 × eye height. Prementum about 0.5 × head height. Labellum developed, about 0.1x as long as prementum.

**Thorax** (Fig. 1a, c): Acrostichal setae 3 + 3 (first presutural seta weak). Dorsocentral setae 2 + 2. Prosternum setulose. Setulose. *Wing*. Costal spine poorly developed. Vein R_4+5_ with setulae dorsally beyond vein r-m and ventrally at base. *Legs*. Fore femur with posterodorsal and posteroventral rows of setae; fore tibia with 7 median anterodorsal, 1 posteroventral in distal third, 3 preapical, 2 anterodorsal and 1 posteroventral setae. Mid tibia with 4 anteroventral, 4 posteroventral setae on apical third; mid femur with anterodorsal setae on apical third, 2 preapical, and 2 posteroventral setae. Hind femur with posterodorsal and posteroventral rows of setae. Hind tibia with rows of anterodorsal (6) and posterodrosal setae (6), 3 submedian posteroventral, 4 preapical, 2 anterodorsal, and 2 posteroventral setae.

**Abdomen** (Fig. 1a, c): Syntergite 1 + 2 with mid-dorsal longitudinal depression extending until ¼ to posterior margin. Syntergite 1 + 2 with at least 4 pairs of lateral marginal setae; tergite 3 with at least 4 pairs of lateral marginal setae and a pair of median marginal seta.

**Terminalia** (Fig. 2): Sternite 5 with slightly developed lobules, setulose, with basal plate long and slightly curved (Fig. 2a, c); sensilla “*trichodea*” present on distal portion. Epandrium broad in posterior view, setulose, and closed dorsally. Surstylus somewhat narrow, not fused with epandrium, convex, setulose in posterior view and with eight short spines laterally on frontal view; distally tapered in lateral view. Extension of dorsal sclerite of distiphallus ending in an expanded region.

**Female**. Differs from male as follows: head with fronto-orbital plate about twice larger as the male, two proclinate and two reclinate orbital setae. Abdomen shorter and broader than male.

**First instar larvae.** A complete description was given by Thompson (1963: 480), and the reader is referred to that work.

**Biology.** Parasitoid of Coleoptera larvae. A specimen from MZSP is pinned with a larva of undetermined species of Hispinae (Chrysomelidae) with a puparium of *E. claripennis* inside it.

**Distribution.** Mexico (Veracruz, new record), Trinidad & Tobago and Brazil (Rio de Janeiro, new record).

***Ebenia fumata*** (Wulp 1891).

(Fig. 3).

**Fig. 3:**
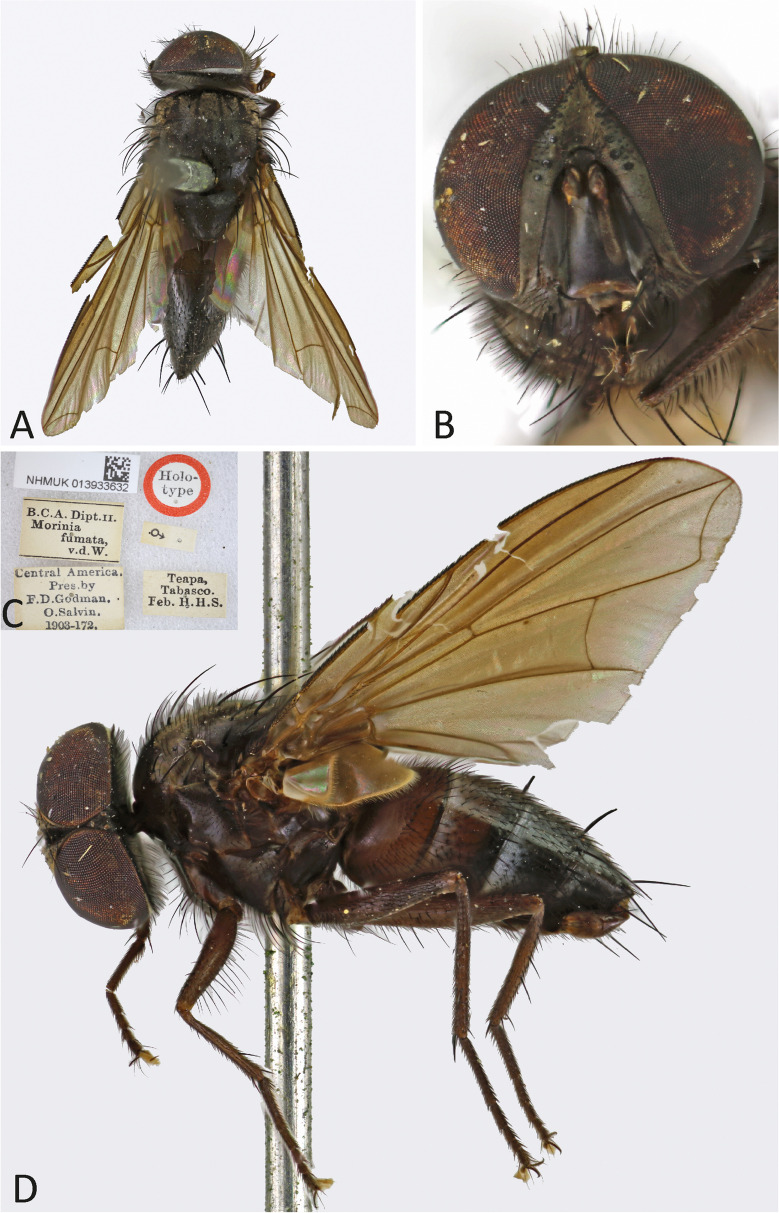
*Ebenia fumata* (Wulp 1891), holotype male (NHMUK). **A**. Dorsal habitus. **B**. head, frontal view. **C**. labels; D. lateral habitus

*Morinia fumata* Wulp 1891: 261. *References*. Guimarães 1971: 110, catalogue – as an unplaced species of “Ebeniini”); O’Hara et al. (2020: 93, checklist of World Tachinidae – new combination as *Ebenia*).

### Type material examined

Holotype ♂: “Holo-/ type”; “♂”; “Teapa,/ Tabasco./ Feb. H.H.S.”; “Central America./ Pres. by/ F.D.Godman./ O.Salvin./ 1903–172.”; “B.C.A. Dipt.II./ Morinia/ fumata,/ v.d. W.”; “NHMUK 013933632”. Holotype in good condition.

### Additional examined material

COSTA RICA. Guanacaste: P.N. Rincón de la Vieja, Sect. Santa Maria, Send. Pailas, Agua Fria, 800 m, 10.xi.2001, 1 ♂, D. Briceño & Libre L., L.N_305475_392908 #65,644 (MNCR).

**Diagnosis.** Eyes with very small and widely scattered setulae. Fronto-orbital plate dark silver pruinose. Postpedicel entirely dark brown. Facial ridge with setulae only at base. Prosternum with setulae. Thorax with silver pruinosity. Wing smoky; with vein R_4+5_ with setulae about ¼ halfway to crossvein r-m. Costal spine poorly developed. Abdomen light brown with silvery pruinosity anteriorly on tergites 3 to 5.

Redescription of holotype male.

**Coloration** (Fig. 3a–b, d): Occiput with silver pruinosity. Head with dark silver pruinosity. Scape light brown and pedicel dark brown. Postpedicel dark brown. Arista dark brown, but proximal 1/5 light brown. Palpus yellowish. Labellum light brown, prementum shiny black. Scutum brownish, but presutural region and anterodorsal portion of postsutural region with brownish-silvery pruinosity; presutural region with five brownish-black vittae, the three central ones narrow and the two peripheral ones broad. Scutellum brownish. Wing smoky on apical region. Tegula and basicosta dark brown. Halter yellowish. Posterior spiracle light-brown. Legs brownish. Upper and lower calypters hyaline. Abdomen light brown with anterolateral silver pruinosity on tergites 3 to 5.

**Head** (Fig. 3a–b, d): Vertex about 0.12 × head width in dorsal view. Width of parafacial, measured at distance between inner margin of eye and antennal insertion, 2 × height of gena. Fronto-orbital plate with setulae throughout its length. Frontal vitta narrowed dorsally. Eye about 0.9 × the head height. Gena about 0.12 × eye height. Prementum about 0.5 × head height. Labellum developed, about 0.1x as long as prementum.

**Thorax** (Fig. 3a, d): Acrostichal setae 3 + 3 (first presutural seta weak). Dorsocentral setae 2 + 2. Prosternum setulose. *Wing*. Costal spine poorly developed. Vein R_4+5_ with setulae dorsally about ¼ halfway to crossvein and ventrally at base. *Legs*. Fore femur with posterodorsal and posteroventral rows of setae; fore tibia with 7 median anterodorsal, 1 posteroventral in distal third, 3 preapical, 2 anterodorsal and 1 posteroventral setae. Mid tibia with 3 anteroventral, 3 posteroventral setae on apical third; mid femur with anterodorsal setae on apical third, 2 preapical, and 2 posteroventral setae. Hind femur with posterodorsal and posteroventral rows of setae. Hind tibia with 2 long submedian anteroventral setae and 2 short apical setae, 4 preapical, 2 anterodorsal, and 2 posteroventral setae.

**Abdomen** (Fig. 3a, d): Syntergite 1 + 2 with mid-dorsal longitudinal depression extending until ¼ to posterior margin. Syntergite 1 + 2 with at least 4 pairs of lateral marginal setae; tergite 3 with at least 4 pairs of lateral marginal setae and a pair of median marginal seta.

**Female**. Unknown.

**Biology**. Unknown.

**Distribution.** Mexico (Tabasco) and Costa Rica (Guanacaste, new record).

***Ebenia neofumata*** Santis & Nihei 2022

(Figs. 4 and 5).

**Fig. 4:**
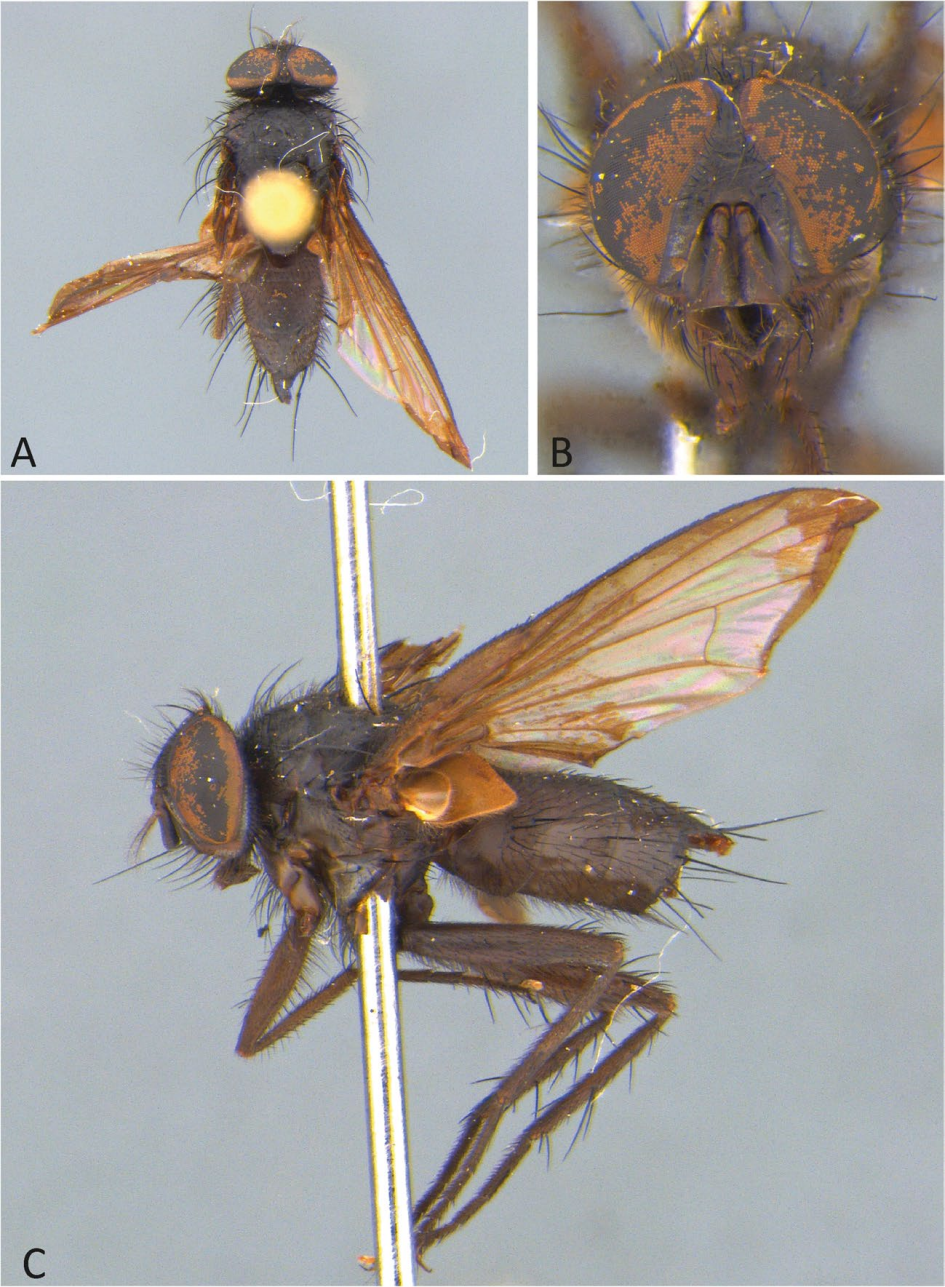
*Ebenia neofumata* Santis & Nihei 2022, male from Guanacaste, Costa Rica. **A**. Dorsal habitus. **B**. head, frontal view. **C**. lateral habitus

**Fig. 5:**
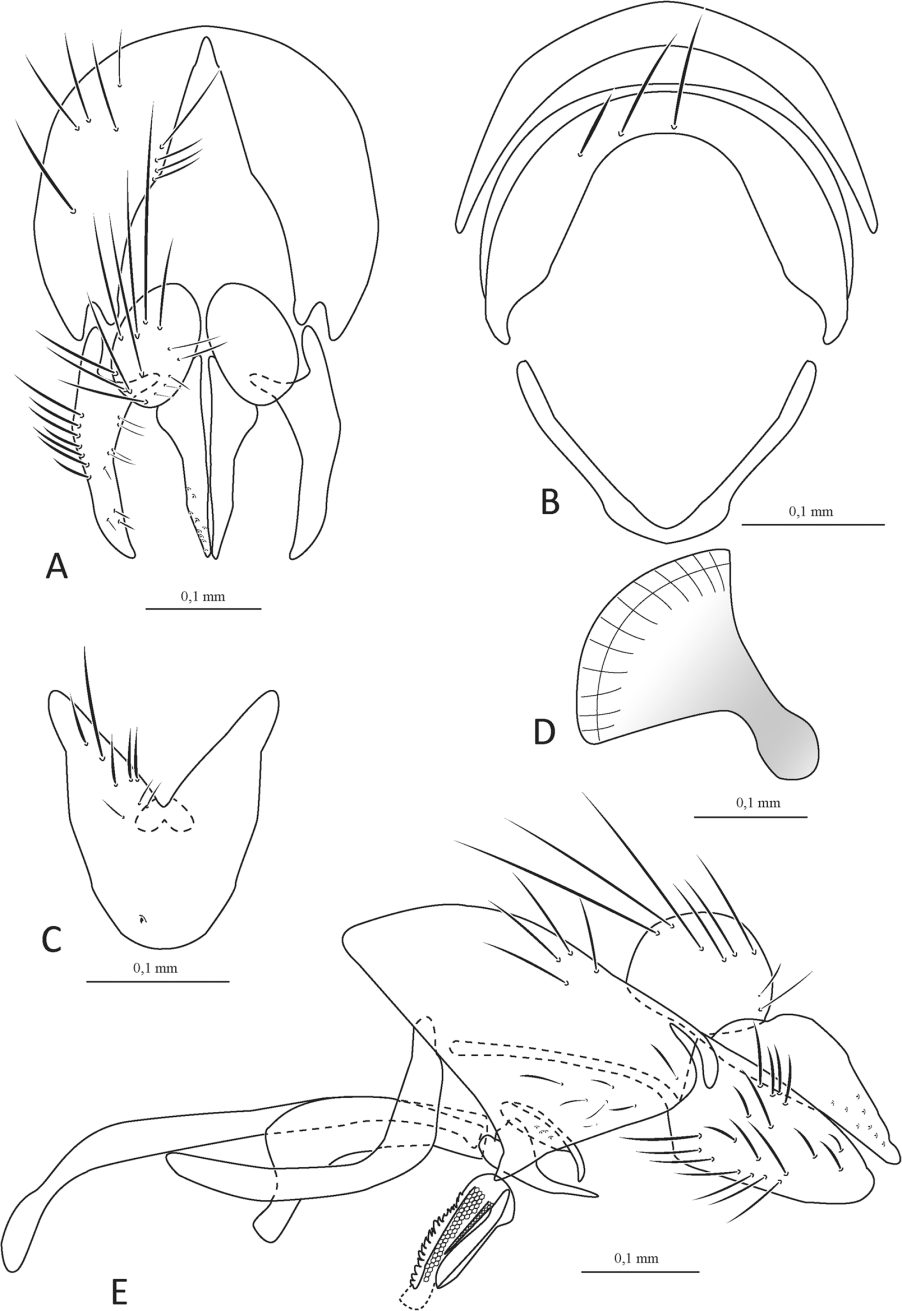
*Ebenia neofumata* Santis & Nihei 2022, Guanacaste, Costa Rica. Male terminalia. **A**. epandrium, cerci and surstylus, posterior view. **B**. syntergosternite 7 + 8, sternite 6 and tergite 6. **C**. sternite 5. **D**. ejaculatory apodeme; **E**. epandrium, cerci, surstylus, hypandrium, phallapodeme, basiphallus, epiphallus, distiphallus, pregonite and postgonite, lateral view

*Ebenia neofumata* Santis & Nihei 2022: 33. *Nomem novum* by Santis & Nihei (2022) for *Comyopsis fumata* Townsend 1919.

*Comyopsis fumata*, Townsend 1919: 176. *References*. Thompson (1963: 475, redescription of male; 477, description of first instar larva); Guimarães 1971: 109, catalogue); Maes (1989: 23, catalogue of parasitoids from Nicaragua); O’Hara et al. (2020: 93, checklist of World Tachinidae); Santis & Nihei (2022, phylogenetic analysis).

### Additional examined material

COSTA RICA. Guanacaste: P.N. Rincón de la Vieja, Sect. Santa Maria, Send. Pailas, Agua Fria, 800 m, 2–5.x.2001, 1 ♂, D. Briceño & Libre L., L.N_305475_392908 #64,949 (MNCR).

**Diagnosis.** Eyes with only a few, scattered short setulae. Fronto-orbital plate dark silver pruinose. Postpedicel entirely dark brown. Facial ridge with setulae only at base. Prosternum bare. Thorax with silver pruinosity. Wing smoky; vein R_4+5_ with setulae ending at the r-m. Costal spine very long, about 3 × the length of adjacent setae. Abdomen blackish, with silver pruinosity only visible on posterior view, occupying about anterior half of tergite 3 and 4. Male terminalia with surstylus setulose in frontal view.

Redescription of male.

**Coloration** (Fig. 4): Occiput with silver pruinosity. Head with dark silver pruinosity. Antenna dark brown. Arista dark brown, but proximal 1/5 light brown. Palpus dark brown. Labellum light brown, prementum shiny black. Scutum brownish, but posterior part of the postpronotal lobe silvery pruinose. Wing smoky. Calypters smoky. Tegula and basicosta dark brown. Halter yellowish-brown. Posterior spiracle light-brown. Legs brownish black. Upper and lower calypters smoky to dark brown. Abdomen blackish, with silver pruinosity only visible on posterior view, occupying about anterior half of tergite 3 and 4.

**Head** (Fig. 4): Vertex about 0.13 × head width in dorsal view. Width of parafacial, measured at distance between inner margin of eye and antennal insertion, 2 × height of gena. Fronto-orbital plate with setulae throughout its length. Frontal vitta narrowed dorsally. Eye about 0.85 × the head height. Gena about 0.12 × eye height. Prementum about 0.5 × head height. Labellum developed, about 0.1x as long as prementum.

**Thorax** (Fig. 4a, c): Acrostichal setae 2 + 1. Dorsocentral setae 2 + 2. Setulose. Anepisternum with one seat on anterior upward region. *Wing*. Costal spine very long, about 3 × the length of adjacent setae. Vein R_4+5_ with setulae ending just at the r-m. *Legs*. Fore femur with posterodorsal and posteroventral rows of setae; fore tibia with a row of anterodorsal setae, 3 preapical, 2 anterodorsal and 1 posteroventral setae. Mid tibia with 5 anteroventral, 5 posteroventral setae on apical third; mid femur with 2 posterodorsal setae and 1 anteroventral setae on distal third, 2 preapical, and 2 posteroventral setae. Hind femur with posterodorsal and posteroventral rows of setae. Hind femur with a row of 8 anteroventral, 8 posteroventral setae, hind tibia with 5 anterodorsal setae, and 1 postodorsal setae on distal third, 2 preapicals, 2 anterodorsal setae.

**Abdomen** (Fig. 4a, c): Syntergite 1 + 2 with mid-dorsal longitudinal depression extending until ½ to posterior margin. Syntergite 1 + 2 with at least 4 pairs of lateral marginal setae; tergite 3 with at least 4 pairs of lateral marginal setae and a pair of median marginal seta.

**Terminalia** (Fig. 5): Sternite 5 with slightly developed lobules, setulose, with basal plate long and slightly curved; sensilla “*trichodea*” present on distal portion. Epandrium broad in posterior view, setulose, and closed dorsally. Anterior epandrial process undeveloped. Cerci not fused, narrow, apically rounded and distally slightly tapered in posterior view. Surstylus somewhat narrow, not fused with epandrium, convex, with seven long setulae in frontal view; distally tapered in lateral view (Fig. 5A, C). Extension of dorsal sclerite of distiphallus ending in an expanded region.

**Female.** Following the description of Thompson (1963: 472), it differs from male by the following: head with vertex about 4x width of front. Ocellar setae weak, proclinate-divergent. A pair of strong proclinate orbitals and between these but near the anterior orbital, a strong reclinate frontal seta.

**First instar larvae.** A complete description was given by Thompson (1963: 477), and the reader is referred to that work.

**Biology.** Unknown.

**Distribution.** Puerto Rico, Trinidad & Tobago, Nicaragua (Chinandega), Mexico (Tabasco) and Costa Rica (Guanacaste, new record).

***Ebenia nigripennis*** (Wulp 1891) **comb. nov.**

(Figs. 6 and 7).

**Fig. 6:**
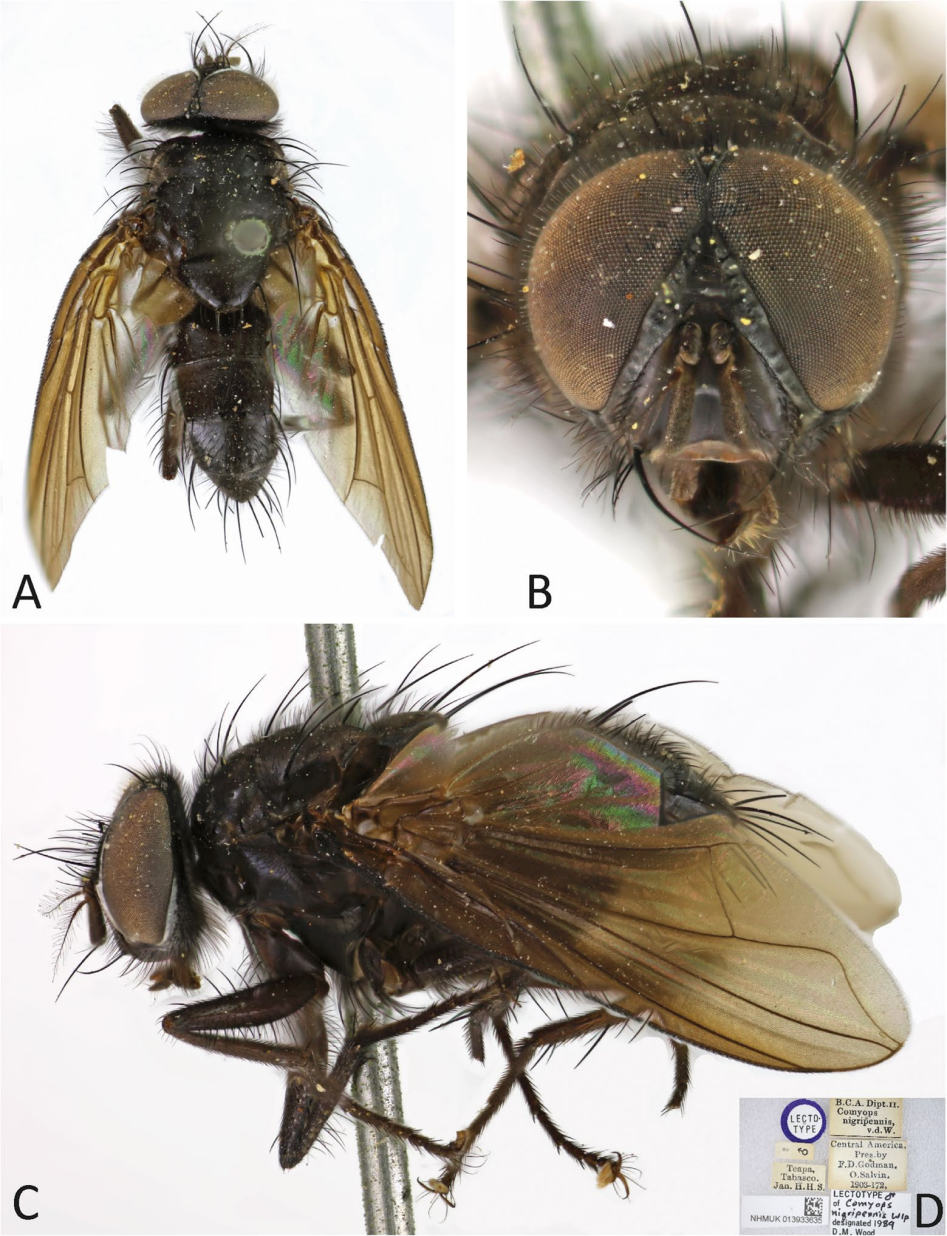
*Ebenia nigripennis* (Wulp 1891), lectotype male (NHMUK). **A**. Dorsal habitus. **B**. head, frontal view. **C**. lateral habitus. d. labels

**Fig. 7:**
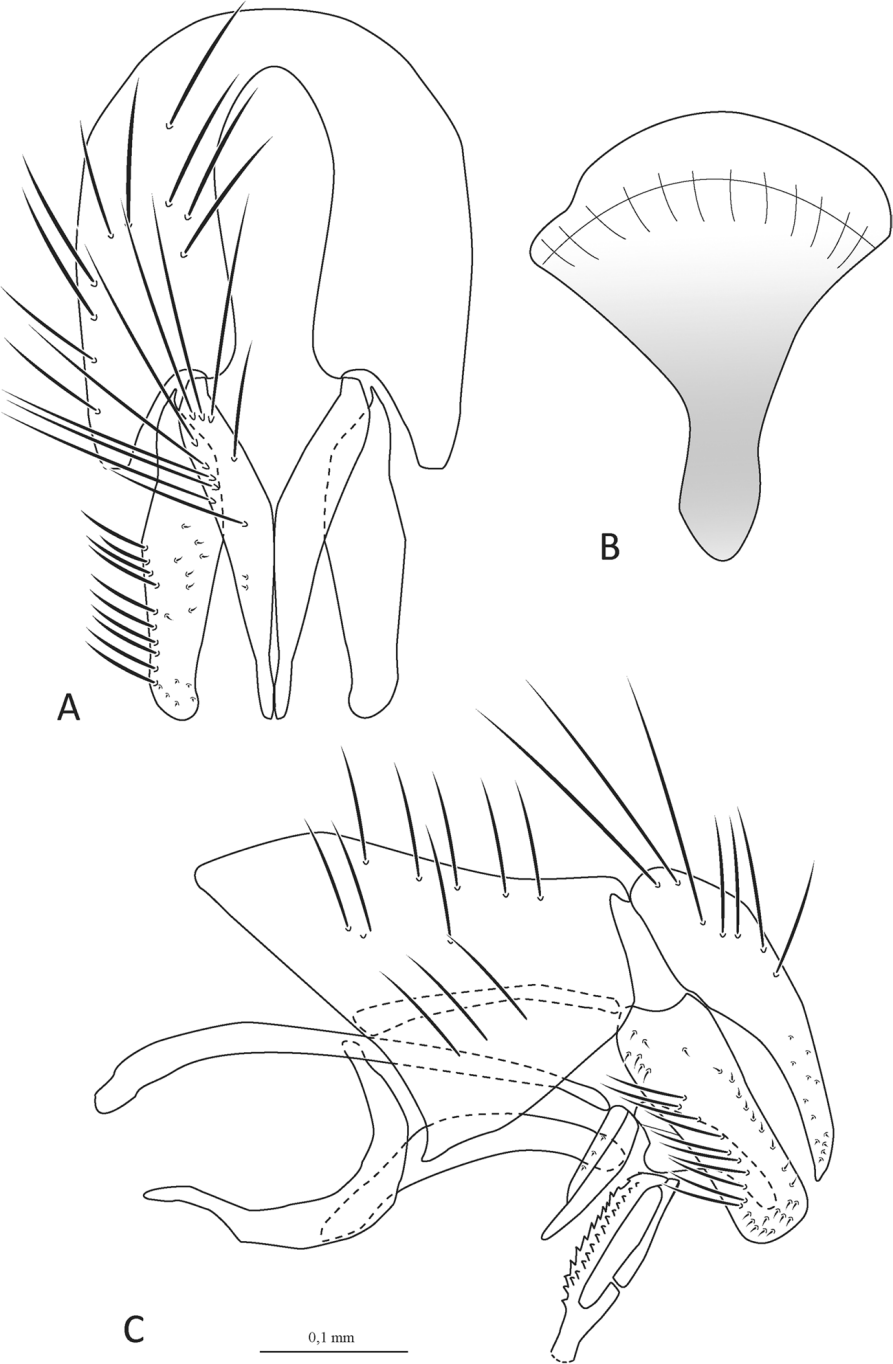
*Ebenia nigripennis* (Wulp 1891), Rio de Janeiro, Brazil (MZSP). Male terminalia. **A**. epandrium, cerci and surstylus, posterior view. **B**. ejaculatory apodeme. **C**. epandrium, cerci, surstylus, hypandrium, phallapodeme, basiphallus, epiphallus, distiphallus, pregonite and postgonite, lateral view

*Comyops nigripennis* Wulp 1891: 262. *References*. Brauer & Bergenstamm (1893: 183, designation of *C*. *nigripennis* as type species of *Comyops*); Thompson (1963: 472, redescription of male; 474, description of first instar larva); Guimarães 1971: 109, catalogue); O’Hara et al. (2020: 91, checklist of World Tachinidae); Santis & Nihei (2022, phylogenetic analysis).

**Remark.** At the NHMUK collection the two male syntypes from the original description of Wulp were examined by D.M. Wood. One male presents a lectotype label and the other male a paralectotype label attached by Wood in 1989. However, the lectotype designation was not published. In the interests of nomenclatural stability and to restrict the name to a single specimen, the male syntype bearing Wood’s lectotype label and the additional label “NHMUK 013933635” is hereby designated as lectotype of *Comyops nigripennis* Wulp 1891.

The current combination for this species is *Ebenia nigripennis* (Wulp, 1891) **comb. nov.**

### Type material examined

Lectotype ♂: “Lecto-/ type”; “♂”; “Teapa,/ Tabasco./ Jan. H.H.S.”; “B.C.A. Dipt.II./ Comyops/ nigripennis,/ v.d. W.”; “Central America./ Pres. by/ F.D.Godman./ O.Salvin./ 1903–172.”; “Lectotype ♂/ of Comyops nigripennis Wlp/ designated 1989/ D. M. Wood”; “NHMUK 013933635”. Lectotype in good condition.

Paralectotype ♂: “Teapa,/ Tabasco./ Jan. H.H.S.”; “B.C.A. Dipt.II./ Comyops/ nigripennis,/ v.d. W.”; “Central America./ Pres. by/ F.D.Godman./ O.Salvin./ 1903–172.”; “Paralectotype ♂/ of Comyops nigripennis Wlp/ designated 1989/ D. M. Wood”.

### Additional examined material

BRAZIL. Rio de Janeiro: Nova Friburgo, Mury, 1 ♂, 1–30.i.1973, Gred & Guimarães col (MZSP).

**Diagnosis.** Eyes with conspicuous setulae. Fronto-orbital plate dark silver pruinose. Postpedicel entirely dark brown. Facial ridge with setulae present almost at the antennal insertion. Prosternum setulose. Thorax without silver pruinosity. Legs brownish black. Wing smoky, mainly on apical portion; vein R_4+5_ with setulae dorsally hallway to r-m. Costal spine poorly developed. Abdomen with silvery pruinosity on each tergite, occupying just anterior margin, about 1/5 of each segment. Male terminalia with surstylus presenting long setulae laterally on frontal view.

Redescription of lectotype male.

**Coloration** (Fig. 6a–c): Occiput with silver pruinosity. Head with dark silver pruinosity. Antenna dark brown. Arista dark brown, but proximal 1/5 light brown. Palpus brownish. Labellum light brown, prementum shiny black. Scutum brownish, but posterior part of the postpronotal lobe silvery pruinose. Wing smoky on apical region. Tegula and basicosta dark brown. Halter yellowish. Posterior spiracle light-brown. Legs brownish. Upper and lower calypters hyaline. Abdomen brownish, with brownish pruinosity only visible on posterior view, occupying about the entire surface of tergite 3 and 4.

**Head** (Fig. 6a–c): Vertex about 0.1 × head width in dorsal view. Width of parafacial, measured at distance between inner margin of eye and antennal insertion, 2 × height of gena. Fronto-orbital plate with setulae throughout its length. Frontal vitta narrowed dorsally. Eye about 0.9 × the head height. Gena about 0.1 × eye height. Labellum developed, about 0.1x as long as prementum.

**Thorax** (Fig. 6a, c): Acrostichal setae 2 + 1. Dorsocentral setae 2 + 3. Prosternum setulose. Anepisternum with one seat on anterior upward region. setulose. *Wing*. Costal spine poorly developed. Vein R_4+5_ with setulae dorsally hallway to vein r-m and ventrally just at base. *Legs*. Fore femur with posterodorsal and posteroventral rows of setae; fore tibia with 1 posteroventral on distal third, 2 preapical, 1 anterodorsal and 1 posteroventral setae. Mid tibia with 3 anteroventral, 3 posteroventral setae on apical third; mid femur with anterodorsal setae on apical third, 2 preapical, and 2 posteroventral setae. Hind femur with posterodorsal and posteroventral rows of setae. hind tibia with 5 anterodorsal setae, and 1 postodorsal setae on distal third, 2 preapicals, 2 anterodorsal setae.

**Abdomen** (Fig. 6a, c): Syntergite 1 + 2 with mid-dorsal longitudinal depression extending until ½ to posterior margin. Syntergite 1 + 2 with at least 4 pairs of lateral marginal setae; tergite 3 with at least 4 pairs of lateral marginal setae and a pair of median marginal seta.

**Terminalia** (Fig. 7): Sternite 5 with slightly developed lobules, setulose, with basal plate long and slightly curved; sensilla “*trichodea*” present on distal portion. Epandrium broad in posterior view, setulose, and closed dorsally. Surstylus somewhat narrow, not fused with epandrium, convex, setulose in posterior view and with about 10 long setulae laterally on posterior view; distally tapered in lateral view (Fig. 7A, C). Extension of dorsal sclerite of distiphallus ending in a confluent region.

**Female**. Following the description of Thompson (1963: 472), it differs from male by the following: head with vertex about 4x width of front. Ocellar setae weak, proclinate-divergent. A pair of strong proclinate orbitals and between these but near the anterior orbital, a strong reclinate frontal seta. Abdominal tergite 1 + 2 with no marginal or discal setae, but a pair of strong lateral setae.

**First instar larvae.** A complete description was given by Thompson (1963: 474), and the reader is referred to that work.

**Biology**. Unknown.

**Distribution.** Mexico, Trinidad & Tobago and Brazil (Rio de Janeiro, new record).

***Ebenia striaticollis*** (Wulp 1891) **comb. nov.**

(Fig. 8).

**Fig. 8:**
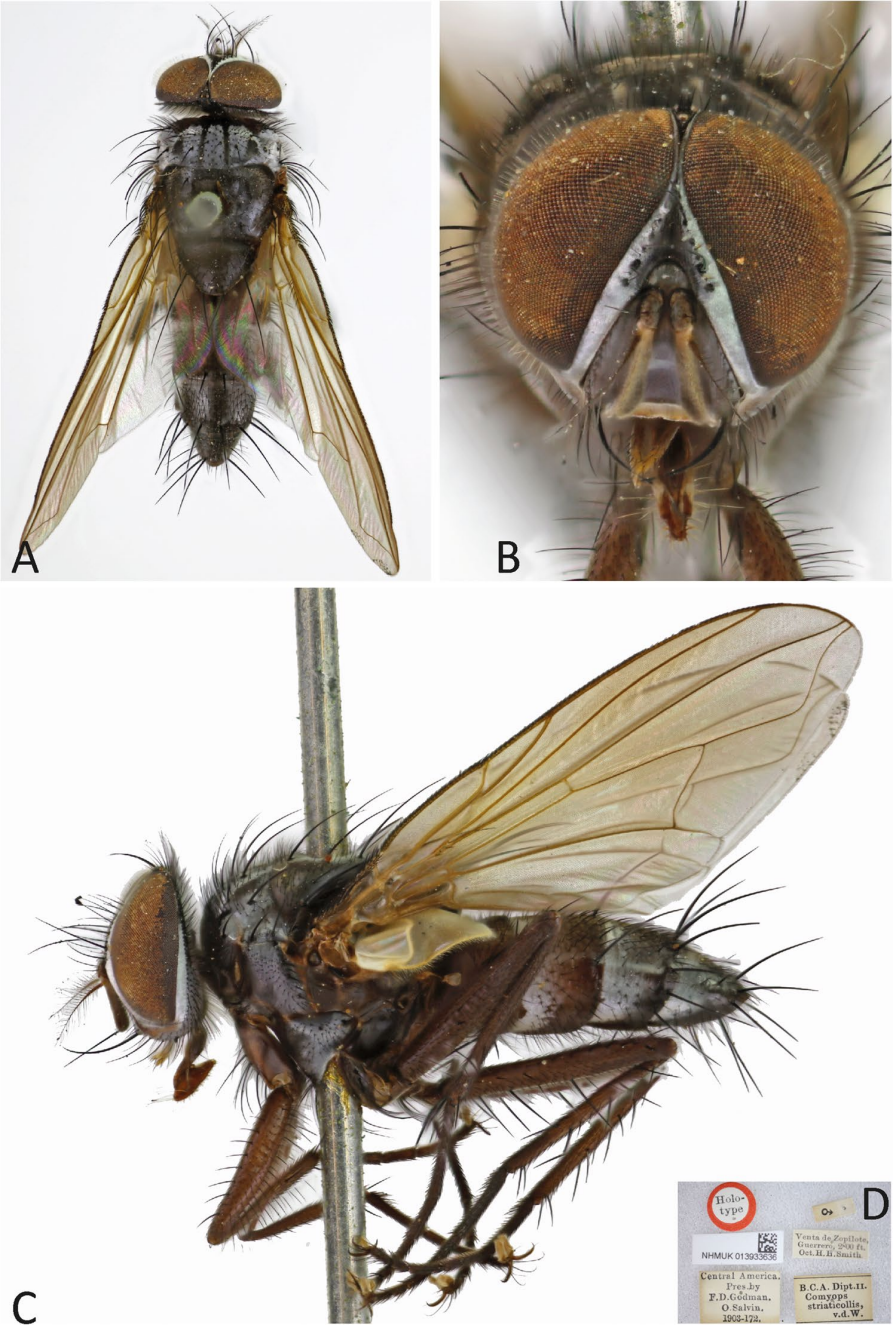
*Ebenia striaticollis* (Wulp 1891), holotype male (NHMUK). **A**. Dorsal habitus. **B**. head, frontal view. **C**. lateral habitus. **D**. labels

*Comyops striaticollis* Wulp 1891: 262. *References*. Guimarães 1971: 109, catalogue); O’Hara et al. (2020: 91, checklist of World Tachinidae).

### Type material examined

Holotype ♂: “Holo-/ type”; “♂”; “Venta de Zopilote,/ Guerrero, 2800 ft,/ Oct. H.H.S.”; “B.C.A. Dipt.II./ Comyops/ striaticollis,/ v.d. W.”; “Central America./ Pres. by/ F.D.Godman./ O.Salvin./ 1903–172.”; “NHMUK 013933636”. Holotype in good condition.

The current combination for this species is *Ebenia striaticollis* (Wulp 1891) **comb. nov.**

**Diagnosis**. Eyes with conspicuous setulae. Fronto-orbital plate silver pruinose. Postpedicel dark brown, but about ¼ dorsal light brown. Facial ridge with setulae present almost at the antennal insertion. Prosternum setulose. Thorax with silver pruinosity. Legs brownish to light brown. Wing hyaline; vein R_4+5_ with setulae dorsally hallway to vein r-m. Costal spine poorly developed. Abdomen with silvery pruinosity on each tergite, almost reaching posterior margin.

Redescription of holotype male.

**Coloration** (Fig. 8a–c): Occiput with silver pruinosity. Head with silver pruinosity. Scape and pedicel brownish. Postpedicel light brown. Arista dark brown, but proximal 1/5 light brown. Palpus yellowish. Labellum light brown, prementum shiny black. Scutum brownish, but presutural region and anterodorsal portion of postsutural region with brownish-silvery pruinosity; presutural region with four brownish-black vittae, the two central ones narrow and the two peripheral ones broad. Wing hyaline. Calypters hyaline. Tegula and basicosta dark brown. Halter yellowish. Posterior spiracle light-brown. Legs brownish. Upper and lower calypters hyaline. Abdomen brownish, with brownish pruinosity reaching about halt the length of each tergite anteriorly.

**Head** (Fig. 8a–c): Vertex about 0.11 × head width in dorsal view. Width of parafacial, measured at distance between inner margin of eye and antennal insertion, 2 × height of gena. Fronto-orbital plate with setulae throughout its length. Frontal vitta narrowed dorsally. Eye about 0.76 × the head height. Gena about 0.13 × eye height. Prementum about 0.5 × head height. Labellum developed, about 0.1x as long as prementum.

**Thorax** (Fig. 8a, c): Acrostichal setae 2 + 2. Dorsocentral setae 2 + 3. Prosternum setulose. Anepisternum with one seat on anterior upward region. *Wing*. Costal spine poorly developed. Vein R_4+5_ with setulae dorsally hallway to vein r-m and ventrally just at base. *Legs*. Fore femur with posterodorsal and posteroventral rows of setae; fore tibia with 1 posteroventral on distal third, 2 preapical, 1 anterodorsal and 1 posteroventral setae. Mid tibia with 4 anteroventral, 4 posteroventral setae on apical third, 6 anterodorsal seta on distal third; mid femur with anterodorsal setae on apical third, 2 preapical, and 2 posteroventral setae. Hind femur with 3 posterodorsal and 3 posteroventral and 2 preapicals, 1 anterodorsal and 1 posterodorsal setae.

**Abdomen** (Fig. 8a, c): Syntergite 1 + 2 with mid-dorsal longitudinal depression extending until ½ to posterior margin. Syntergite 1 + 2 with at least 4 pairs of lateral marginal setae; tergite 3 with at least 4 pairs of lateral marginal setae and a pair of median marginal seta.

**Female**. Unknown.

**Biology**. Unknown.

**Distribution.** Mexico (Guerrero).

**Fig. 9:**
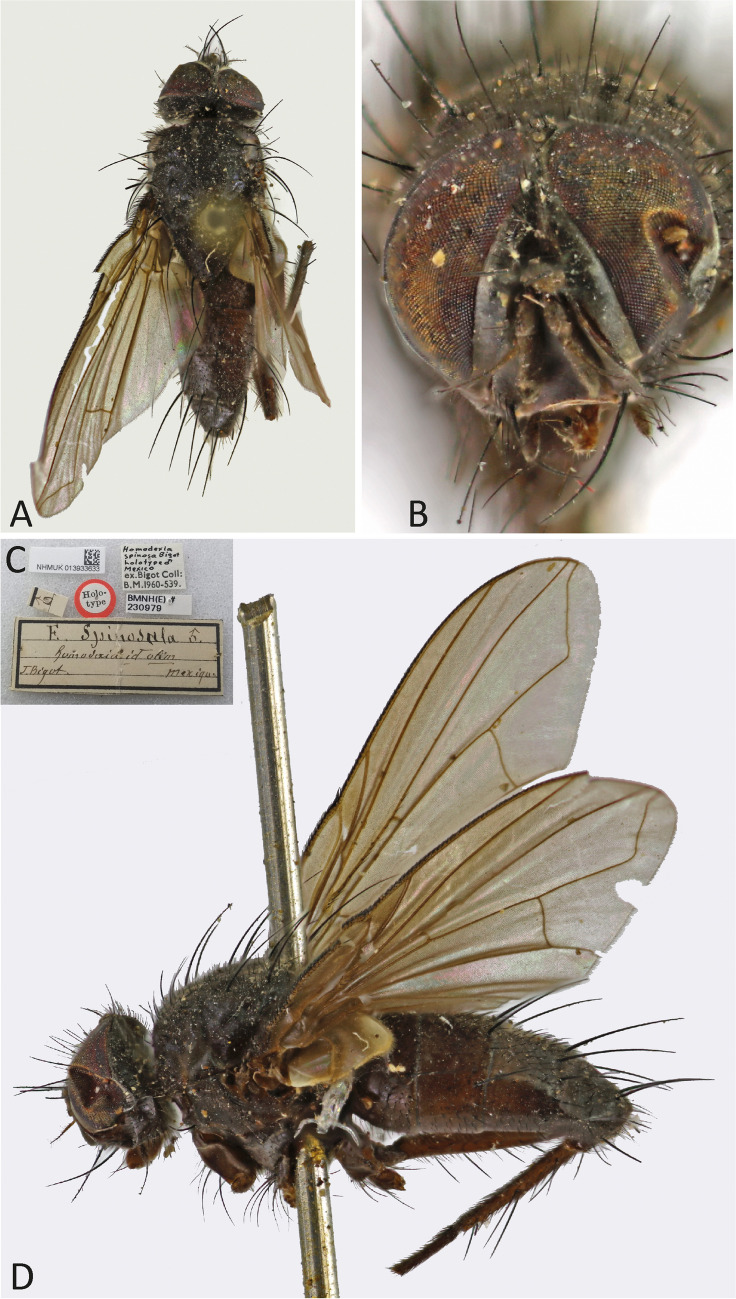
*Ebenia spinosa* (Bigot 1889), holotype male (NHMUK). **A**. Dorsal habitus. **B**. head, frontal view. **C**. labels. **D**. lateral habitus

**Fig. 10:**
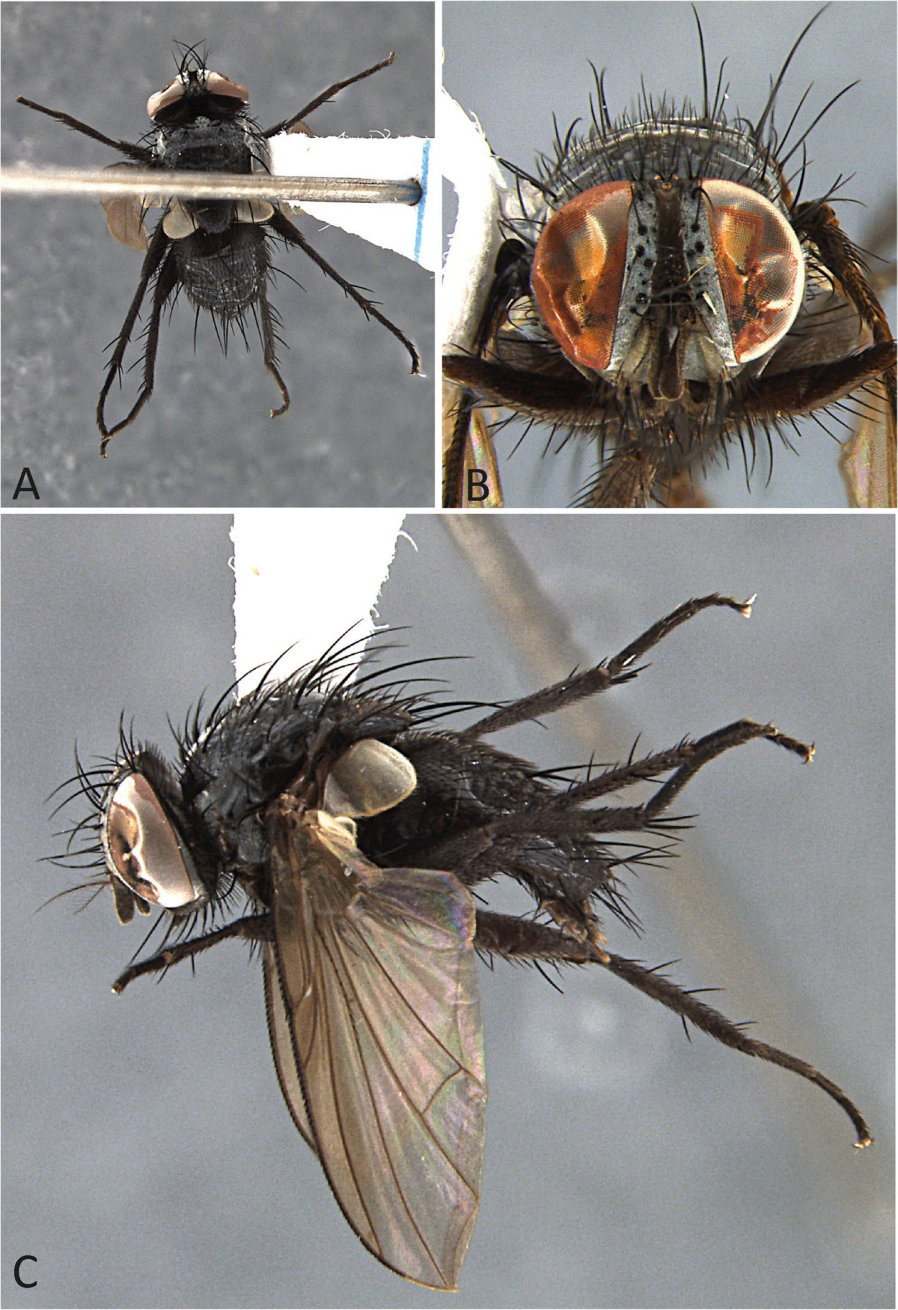
*Ebenia spinosa* (Bigot 1889), female from Rio de Janeiro, Brazil (MNRJ). **A**. Dorsal habitus. **B**. head, frontal view. **C**. lateral habitus

**Fig. 11:**
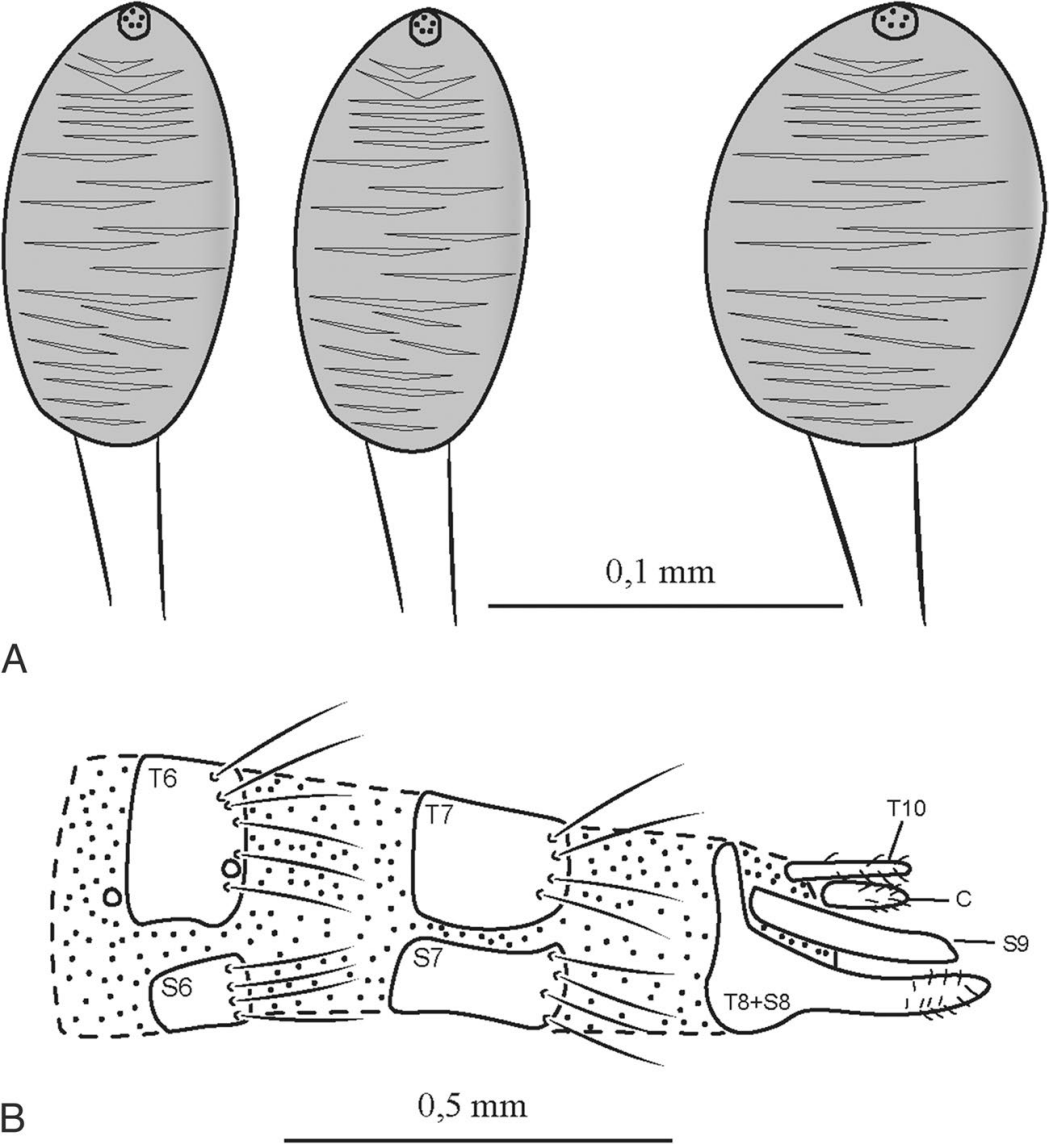
*Ebenia spinosa* (Bigot 1889), Cartago, Costa Rica (MNCR). **A**. Spermathecae. **B**. female terminalia. Abbreviations: C = cercus; S = sternite; T = tergite

***Ebenia spinosa*** (Bigot 1889) **comb. nov.**

(Figs. 9, 10 and 11).

*Homodexia spinosa* Bigot 1889: 268. Male holotype (NHMUK). Type locality: Mexico. *Reference*. Wulp 1891: 264, diagnostic traits; close resemblance with *Morinia* Robineau-Desvoidy, 1830 sensu Wulp, i.e., *Ebenia* in part);

*Ebenia spinosa* (Bigot 1889). *Reference*. Brauer (1898: 515, diagnostic traits; close to *Thelairodes* Wulp 1891);

*Thelairodes spinosus* (Bigot 1889). *References*. Guimarães 1971: 195, catalogue); O’Hara et al. (2020: 174, checklist of World Tachinidae).

*Morinia trichopoda* Wulp 1891: 261. Mexico, Veracruz and Tabasco. Two males syntypes and a single female syntype (NHMUK). **New synonymy**

*Ebenia trichopoda* (Wulp 1891). *References*. Guimarães 1971: 109, catalogue); O’Hara et al. (2020: 93, checklist of World Tachinidae).

### Type material examined

Holotype ♂ of *Homodexia spinosa* Bigot 1889: “Holo-/ type”; “E. spinosula ♂./ Homodexia id olim/ J. Bigot. Mexique”; “♂”; Homodexia/ spinosa Bigot/ holotype ♂/ Mexico [handwritten]/ ex.Bigot Coll: B.M.1960–539.”; “BMNH(E) #/ 230,979”; “NHMUK 013933633″. Holotype in fair condition, slightly molded; head and wings damaged, only a single hind leg present.

**Remarks**. In the original description of *Morinia trichopoda*, Wulp 1891: 261) mentioned “Six male and two female specimens”, however, at the NHMUK collection, only two males and one female could be found. These three syntypes were examined by D.M. Wood. One male presents a lectotype label and the other specimens a paralectotype label each attached by Wood in 1989. However, the lectotype designation was not published. In the interests of nomenclatural stability and to restrict the name to a single specimen, the male syntype that bears Wood’s lectotype label and the additional label “NHMUK 013933635” is hereby designated as lectotype of *Morinia trichopoda* Wulp 1891.

The current status for this species is a junior synonymy of *Homodexia spinosa* Bigot 1889.

### Type material examined

Lectotype ♂ of *Morinia trichopoda* Wulp 1891: “Lecto-/ type”; “♂”; “Teapa,/ Tabasco./ Jan. H.H.S.”; “B.C.A. Dipt.II./ Morinia/ trichopoda,/ v.d. W.”; “Central America./ Pres. by/ F.D.Godman./ O.Salvin./ 1903–172.”; “Lectotype ♂/ of Morinia/ trichopoda Wlp/ designated 1989/ D. M. Wood”; “NHMUK 013933635”.

*Paralectotype* ♂: “Teapa,/ Tabasco./ March. H.H.S.”; “B.C.A. Dipt.II./ Morinia/ trichopoda,/ v.d. W.”; “Central America./ Pres. by/ F.D.Godman./ O.Salvin./ 1903–172.”; “Paralectotype ♂/ of Morinia/ trichopoda Wlp/ designated 1989/ D. M. Wood”.

*Paralectotype* ♀: “Vera,/ Tabasco./ March. H.H.S.”; “B.C.A. Dipt.II./ Morinia/ trichopoda,/ v.d. W.”; “Central America./ Pres. by/ F.D.Godman./ O.Salvin./ 1903–172.”; “Paralectotype ♀/ of Morinia/ trichopoda Wlp/ designated 1989/ D. M. Wood”.

### Additional examined material

COSTA RICA. Cartago: P.N. Barbilla, 3 km, S.F. de la Estación, 400 m, 16.xii.2000, 1 ♀, E. Rojas, Manual, L.N_216200_598100 #62,968 (MNCR); BRAZIL. Rio de Janeiro: Casimiro de Abreu, Rebo União, Trilha do Buração, malaise, 1 ♀, 8.iii.2014, Eq. col., Biota Diptera Fluminense (MNRJ), ditto, Trilha Três Pontes, 1 ♀, 27.v-27.vi.2013, Eq. col., Biota Diptera Fluminense (MNRJ).

**Diagnosis**. Eyes with conspicuous setulae. Fronto-orbital plate silver pruinose. Postpedicel dark brown, but about ¼ dorsal light brown. Facial ridge with setulae present almost at the antennal insertion. Prosternum setulose. Thorax with silver pruinosity. Legs brownish to light brown. Wing hyaline; vein R_4+5_ with setulae dorsally hallway to vein r-m. Costal spine poorly developed. Abdomen with silvery pruinosity on each tergite, almost reaching posterior margin.

(Figs. 9–11 here).

Redescription of male.

**Coloration** (Fig. 9a–b, d): Occiput with silver pruinosity. Head with dark silver pruinosity. Scape light brown and pedicel dark brown. Postpedicel dark brown. Arista dark brown, but proximal 1/5 light brown. Palpus yellowish. Labellum light brown, prementum shiny black. Scutum brownish, but presutural region and anterodorsal portion of postsutural region with brownish-silvery pruinosity; presutural region brownish-black, with a silver pruinosy median vittae. Wing smoky on apical region. Tegula and basicosta dark brown. Calypters smoky. Halter yellowish. Posterior spiracle light-brown. Legs brownish. Upper and lower calypters hyaline. Abdomen brownish, without pruinosity.

**Head** (Fig. 9a–b, d)*:* Vertex about 0.14 × head width in dorsal view. Width of parafacial, measured at distance between inner margin of eye and antennal insertion, 2 × height of gena. Fronto-orbital plate with setulae throughout its length. Frontal vitta narrowed dorsally. Eye about 0.8 × the head height. Gena about 0.16 × eye height. Labellum developed, about 0.1x as long as prementum.

**Thorax** (Fig. 9a, c): Acrostichal setae 2 + 1 (first presutural seta weak). Dorsocentral setae 1 + 2. Prosternum setulose. Anepisternum with one seat on anterior upward region. *Wing*. Costal spine well developed. Vein R_4+5_ with setulae beyond the r-m and ventrally at base. *Legs*. Fore femur with posterodorsal and posteroventral rows of setae; fore tibia with 7 median anterodorsal, 1 posteroventral in distal third, 5 preapicals, 2 anterodorsal, 1 lateral and 2 posteroventral setae. Mid tibia with 3 anteroventral, 3 posteroventral setae on apical third; mid femur with anterodorsal setae on apical third, 2 preapical, and 2 posteroventral setae. Hind femur with posterodorsal and posteroventral rows of setae. Hind tibia with 2 long submedian anteroventral setae and 2 short apical setae, 4 preapical, 2 anterodorsal, and 2 posteroventral setae.

**Abdomen** (Fig. 9a, c): Syntergite 1 + 2 with mid-dorsal longitudinal depression extending until ½ to posterior margin. Syntergite 1 + 2 with at least 4 pairs of lateral marginal setae; tergite 3 with at least 4 pairs of lateral marginal setae and a pair of median marginal seta.

**Female**. Differs from male as follows (Fig. 10): Head vertex about 0.21 × head width in dorsal view, with fronto-orbital plate with 1 proclinate, followed apically by 1 reclinate and other 1 proclinate pairs of setae. Frontal vittae equal length from the vertex, viewed dorsally. Legs with claws and pulvilli shorter than tarsomere 5. Abdomen shorter and broader than in the male, brownish black.

**Terminalia** (Fig. 11b): Tergites 6–7 and sternites 6–7 as a subrectangular plate with setae posteriorly. Tergite 8 fused with sternite 8, forming a cone shape (posteriorly facing) structure. Sternite 9 well-developed and long. Sternite 10 long, rod-shaped, with setulae along its structure. Cercus subovalate with setulae posteriorly. Spermatheca (Fig. 11a): two narrow, subovalate and one ovalate, broader; slightly rugose with a pore apically.

**Biology**. Unknown.

**Distribution**. Mexico (Tabasco); Costa Rica (Cartago, new record); Brazil (Rio de Janeiro, new record).

**Remarks**. Jacques-Marie-Frangile Bigot (1818–1893) was a French dipterist who accumulated an exceptionally rich collection of material from the Neotropical region over the years. Mexico was frequently cited for type localities, enabling the description of numerous new species. However, there are two main problems with his species: (1) because of Bigot’s brief and uninformative descriptions, “…without reference to the type specimens it is virtually impossible to recognize any of the genera and species which Bigot described…” (Crosskey 1971: 293) and (2) for his careless regard for nomenclature and orthography (see, e.g., Verrall 1889). The holotype of this species is a fine example of Bigot’s procedure. One can read at the original label of the holotype male (Fig. 9D), by the handwriting of Bigot himself, “*Homodexia id olim*”; “id.” meaning idem and “olim” for formerly, i.e., equal to the formerly genus. In addition, Bigot wrote “*E. spinosula*”, and not *Homodexia spinosa*. Thus, Bigot, since the original description, probably had changed his mind and preferred to consider this species as belonging to *Ebenia* and not *Homodexia*, as Brauer (1898) noted. Additionally, Wulp 1891: 264) considered *Homodexia spinosa* with a close resemblance with *Morinia* (= *Ebenia* in part, sensu Wulp). On the other hand, Brauer (1898) considered that the characters of this species agree with the genus *Thelairodes* Wulp 1891. Probably following Brauer (1898), Guimarães (1971) placed this genus as belonging to *Thelairodes*, a placement followed by O’Hara et al. (2020). Herein, I could confirm Bigot’s change of mind as seen in his label by showing that *Homodexia spinosa* is indeed a species pertaining to *Ebenia* as the diagnostic characters above shows.

Additional complexities for anyone studying Bigot’s material are that his type-specimens are normally only known to the country and he never cites the names of the collectors. Most of the insects collected in Mexico, especially for the nineteenth century, were acquired in Europe through professional collectors, who explored Mexico for many years, visiting almost every state of the country. Thus, Papavero (1971) considered very likely that Bigot studied the collections gathered in Mexico by Louis Pilate (1816–1852), a French naturalist; by three naturalists that belonged to a Belgian commission charged by the government to undertake a scientific exploration that included Mexico: August Boniface Ghiesbreght (1810–1893), a Belgian zoologist; Jean Jules Linden (1817–1898), a Belgian botanist, and Nicholas Funck (1817–1896) a Luxembourgish, the artist of the expedition. Additionally, the naturalists, that are also known for sending part of their collections to Luigi Bellardi (1818–1889) in Italy, could also have been used by Bigot in his works: Auguste Sallé (1820–1896) a French traveler, Adrien Jean Louis François Sumichrast (1828–1882) a Swiss naturalist and professional collector, Henri Louis Frédéric de Saussure (1829–1905), a Swiss mineralogist and entomologist, and Adolphe Boucard (1839–1905) a French ornithologist and collector. Thus, it is virtually impossible to state a precise locality for *E. spinosa* without at least the information of who, among those distinguished travelers and naturalists that visited different regions of Mexico, was the one that collected this specimen.

## References

[CR1] Bigot J-M-F (1889). Diptères nouveaux ou peu connus 34e partie. XLII. Diagnoses de nouvelles espèces. Ann Soc Entomol Fr Sér.

[CR2] Brauer F (1898) Beiträge zur Kenntniss der Muscaria schizometopa. I. Bemerkungen zu den Originalexemplaren der von Bigot, Macquart und Robineau-Desvoidy beschriebenen Muscaria schizometopa aus der Sammlung des Herrn G.H. Verrall. Zweite Folge. Sitz.-Ber. K. Akad. Wiss. Math.-Naturwiss. Kl. Abteilung I 107:493–546

[CR3] Brauer F, Bergenstamm JE von (1893) Die Zweiflügler des Kaiserlichen Museums zu Wien. VI. Vorarbeiten zu einer Monographie der Muscaria schizometopa (exclusive Anthomyidae). Pars III. Denkschrift der Akad der Wissenschaften Wien 60:89–240

[CR4] Brauer F, von Bergenstamm JE (1891). Die Zweiflügler des Kaiserlichen Museums zu Wien. V.Vorarbeiten zu einer Monographie der Muscaria Schizometopa (exclusive Anthomyidae). Pars II. Denkschrift Der Akad Der Wissenschaften Wien.

[CR5] Crosskey RW (1971). The type-material of Australasian, Oriental and Ethiopian Tachinidae (Diptera) described by Macquart and Bigot. Bull Nat Hist Mus, Entomol.

[CR6] Cuignet M, Windsor MD, Reardon J, Hence T, Jolivet PH, Santiago-Blay JA, Schmitt M (2008). The diversity and specificity of parasitoids attacking Neotropical tortoise beetles (Chrysomelidae: Cassidinae). Research on Chrysomelidae.

[CR7] Cumming JM, Wood DM (2017) Adult morphology and terminology. In: Kirk-Spriggs AH, Sinclair BJ (eds) Manual of afrotropical diptera, vol 1. Suricata 4, Pretoria, South African National Biodiversity Institute Graphics & Editing, pp 89–133

[CR8] Evenhuis NL, Pape T, Pont AC (2016). Nomenclatural studies toward a world list of Diptera genus-group names Part V: Pierre-Justin-Marie Macquart. Zootaxa.

[CR9] Guimarães JH (1971) Family tachinidae (Larvaevoridae). In: Papavero N (ed) A catalogue of the diptera of the Americas South of the United States, vol 104, pp 1–333. Museu de Zoologia, Universidade de São Paulo, São Paulo

[CR10] Janzen DH, Hallwachs W (2005) Dynamic database for an inventory of the macrocaterpillar fauna, and its food plants and parasitoids of the Area de Conservacion Guanacaste (ACG), northwestern Costa Rica. Available from URL: http://janzen.sas.upenn.edu. Accessed 1 September 2023

[CR11] Macquart J (1846a) Diptères exotiques nouveaux ou peu connus. [1.er] Supplément. Mem Soc Sci Agric Lille 133–364+20 pls

[CR12] Macquart J (1846b) Diptères exotiques nouveaux ou peu connus. Supplément, Paris 5–238 + 20 pls

[CR13] Maes JM (1989). Catálogo de los insectos controladores biológicos en Nicaragua Volumen III Insectos parasitoides. Rev Nica Ent.

[CR14] O’Hara JE, Henderson SJ (2020) World genera of the Tachinidae (Diptera) and their regional occurrence. Version 1.1. PDF document, 90 pp. Available from URL: http://www.nadsdiptera.org/Tach/WorldTachs/Genera/Worldgenera.htm. Accessed 1 October 2023

[CR15] O’Hara JE, Henderson SJ, Wood DM (2020) Preliminary checklist of the Tachinidae (Diptera) of the world. Version 2.1. PDF document, 1039 pp. Available from URL: http://www.nadsdiptera.org/Tach/WorldTachs/Checklist/Worldchecklist.html. Accessed 1 October 2023

[CR16] Papavero N (1971). Essays on the history of Neotropical Dipterology, with special reference to collectors (1750–1905).

[CR17] Santis MD (2021). The Neotropical genus *Eudexia* Brauer & Bergenstamm, 1889 (Dexiini, Dexiinae): lectotype fixation for the type species, synonymy, and a new species from Brazil. Zootaxa.

[CR18] Santis MD (2022). A bibliographic review of the history of Dexiinae (Diptera, Tachinidae) taxonomy in the Neotropical Region with bibliographic notes on Dominik Bilimek and Fritz Plaumann. Arqu Zool.

[CR19] Santis MD, Nihei SS (2022). Phylogenetic analysis of the tribe Dufouriini (Diptera, Tachinidae) using a total evidence approach based on adult and immature stages. Arthropod Syst Phylogeny.

[CR20] Stireman JO, Cerretti P, O’Hara JE, Blaschke JD, Moulton JK (2019). Molecular phylogeny and evolution of world Tachinidae (Diptera). Mol Phylogenet Evol.

[CR21] Thompson WR (1963). The Tachinids of Trinidad. II. Echinomyiines, Dexiines, and allies. Can J Zool.

[CR22] Townsend CHT (1893). I.-Catalogue of the described south american species of calyptrate muscidae. Ann N Y Acad Sci.

[CR23] Townsend CHT (1919). New muscoid genera, species and synonymy (Diptera). [Concl.] Insecutor Inscitiae Menstruus.

[CR24] Townsend CHT (1936) Manual of myiology in twelve parts. Part IV. Oestroid classification and habits.Dexiidae and Exoristidae. Privately published, Itaquaquecetuba, São Paulo

[CR25] Townsend CHT (1939) Manual of myiology in twelve parts. Part IX. Oestroid generic diagnoses and data.Thelairini to Clythoini. Privately published, Itaquaquecetuba, São Paulo

[CR26] Verbeke J (1962). Contribution a l’étude des Tachinidae africains (Diptera). Exploration Hydrobiologique des Lacs Kivu, Édouard et Albert (1952–1954). Résultats scientifiques.

[CR27] Verrall GH (1889). Bigot’s orthography. Wien Ent Ztg.

[CR28] Wood DM, Zumbado MA (2010) Tachinidae (tachinid flies, parasitic flies). In: Brown BV, Borkent A, Cumming JM, Wood DM, Woodley NE, Zumbado MA (eds) Manual of central american diptera, vol 2. NRC Research Press, Ottawa, pp 1343–1417

[CR29] Wulp FM van der (1891) Fam. Muscidae. pp. 249–264 + pls. 5–6. [Cont.] In: Godman FD, Salvin O (eds) Biologia Centrali-Americana, or, contributions to the knowledge of the fauna and flora of Mexico and Central America. Zoologia. Class Insecta. Order Diptera. Vol. II. [1888–1903] Taylor & Francis, London

